# Case report: septic shock after endometrial polypectomy with tissue removal system

**DOI:** 10.1186/s12905-023-02690-9

**Published:** 2023-10-23

**Authors:** Danna Su, Jiajie She, Yuting Xu, Ying Li, Yan Guo, Yajie Yang, Qiao Tan, Liping Wang, Ruiying Diao

**Affiliations:** 1 Reproductive Medicine Centre, The First Affiliated Hospital of Shenzhen University, Shenzhen Second People’s Hospital, Shenzhen, 518035 China; 2 Shantou University Medical College, Shantou, Guangdong China; 3 Department of Pathology, The First Affiliated Hospital of Shenzhen University, Shenzhen Second People’s Hospital, Shenzhen, China

**Keywords:** Hysteroscopy, Hysteroscopic tissue removal system, Endometrial polypectomy, Postoperative complications, Shock, Septic

## Abstract

As an emerging surgical technology, tissue removal systems have been widely used in the treatment of endometrial polyps due to its characteristics of less endometrial damage, shorter learning curve and clearer vision of the operative field. There are few cases in the literature reporting serious complications after endometrial polypectomy using tissue removal systems. As known, septic shock is a rare complication following hysteroscopic polypectomy. Now, we present the case of a 23-year-old woman who developed septic shock after polypectomy with tissue removal system. The patient had a history of recurrent vaginitis for more than half a year. Due to endometrial polyps, she was admitted to our hospital and scheduled to undergo hysteroscopic endometrial polypectomy. Three hours after the endometrial polypectomy using the tissue removal system, the patient had shock symptoms such as increased body temperature, decreased blood pressure and increased heart rate. Then, the patient was successfully treated and discharged after anti-infection and anti-shock treatments. The purpose of this case report is to remind clinicians to consider the possibility of serious infection and comprehensively evaluate the risk of infection before choosing hysteroscopic devices for endometrial polyps, especially for patients who choose the mechanical hysteroscopic tissue removal systems. Furthermore, the mechanical hysteroscopic tissue removal systems should be used with caution in patients with previous recurrent vaginitis.

## Introduction

Endometrial polyps (Eps) represent a frequent benign focal overgrowth of endometrial mucosa. The formation of Eps is related to the high level of estrogen [1]. Other risk factor including age, hypertension, obesity, and tamoxifen use have been associated with the development of Eps [2]. Eps can lead to abnormal uterine bleeding and infertility, while the potential for malignant transformation of untreated Eps is unknow [2]. However, hysteroscopic surgery is recommended for large and symptomatic polyps [3].

Hysteroscopic electrosurgical techniques are the commonest method to remove Eps [4]. The complication rate of hysteroscopic polypectomy is low, only about 0.22% [5]. As a new surgical device emerging in recent years, the mechanical hysteroscopic tissue removal systems have significantly shortened operation time and improves success rate of surgery while not increasing complication rates [4, 6, 7]. Herein, we reported a patient with a history of recurrent vaginitis who suffered septic shock after endometrial polypectomy with the tissue removal system. This is the first case report of serious complication after hysteroscopic morcellation.

## Case presentation

On June 29, 2022, a 23-year-old young woman with a BMI of 18.2 kg/m^2^ was admitted to our hospital because of Eps found more than half a year ago. The patient lacked exercise at ordinary times and denied previous history of surgery as well as chronic diseases in the past. The examination of ovarian reserve function in another hospital found that AMH was low (less than 1 ng/ml), indicating primary ovarian insufficiency. She had regular menstruation and had never been pregnant. No special family history of disease was identified in this patient.

More than half a year ago, the uterine ultrasound examination of this patient revealed the presence of hyperechoic lesions with smooth and regular contours within the uterine lumen. These lesions were encircled by thin hyperechoic haloes. These findings are indicative of endometrial polyps. Meanwhile, vaginal discharge were positive for mycoplasma, bacteria and fungi. The vaginal discharge was performed again after treatment with "nifuratel nystatin vaginal soft capsules and doxycycline hyclate tablets", and was negative for mycoplasma and bacteria but still positive for fungi. The patient was then treated with "clotrimazole tablet" for 4 times. However, her subsequent vaginal discharge still showed positive for fungus. Luckily, the fungus finally turned negative after receiving the treatment of "fluconazole tablets". Therefore, the patient was admitted to the hospital for hysteroscopic surgery.

Following admission, a series of testing projects were conducted including blood routine, coagulation function, liver and kidney function, preoperative assessment for infectious diseases, electrocardiogram, and chest X-ray. No significant abnormalities were found. Given the patient had fertility requirements, she met the inclusion criteria of a clinical study being conducted in our hospital (clinical trial numbers: ChiCTR2200058712). After signing informed consent, the patient was recruited into the study (ethical review approval number: 20210620213357026-FS01). In addition, the patient required tubal hydrotubation during the operation because she failed in trying to conceive for more than half a year.

**Fig. 1:**
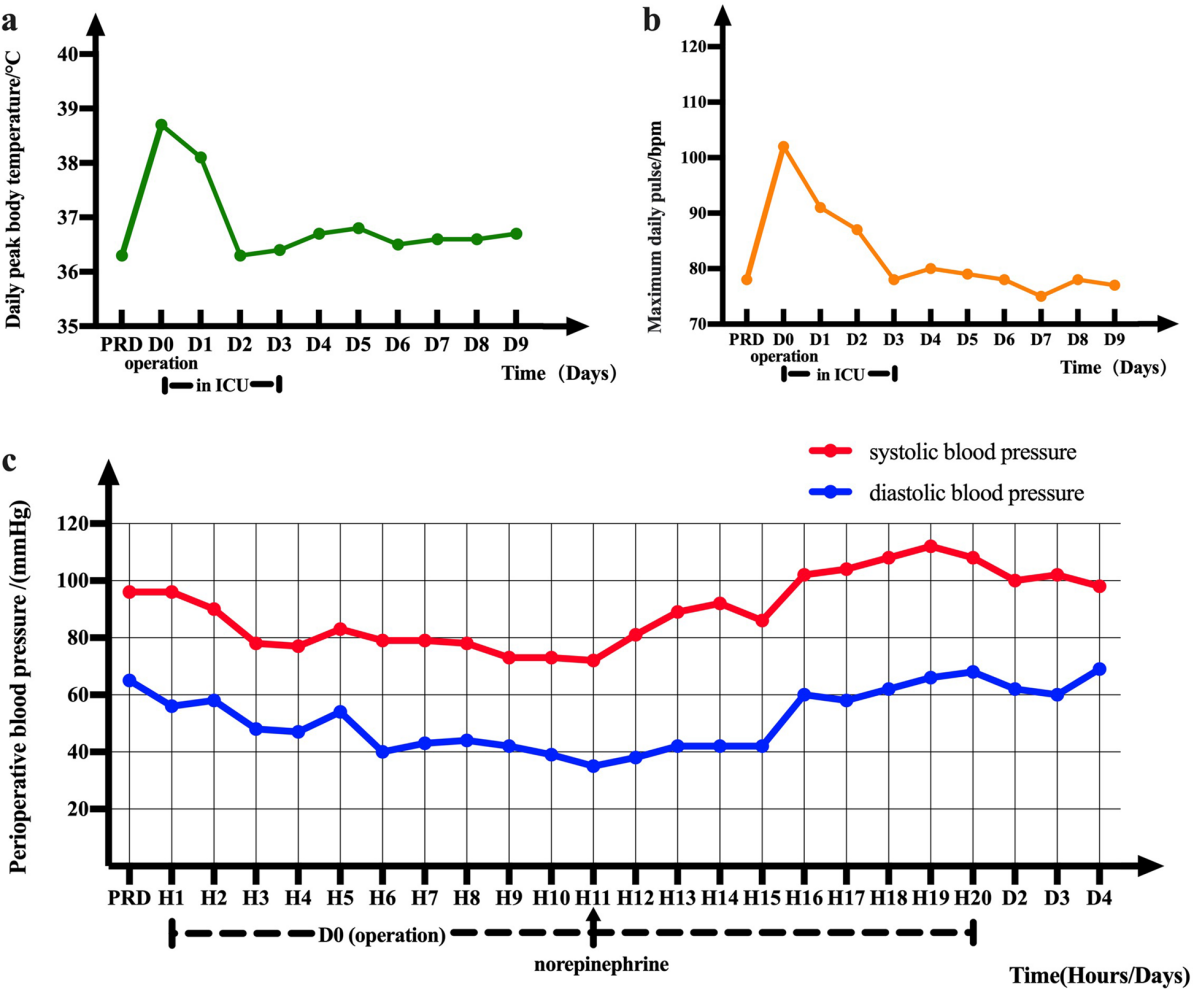
Line Chart Illustrating Patient's Vital Signs During Hospitalization. **a** Temperature during hospitalization. **b** Daily pulse during hospitalization. **c** Perioperative changes in systolic blood pressure (SBP) and diastolic blood pressure (DBP). * PRD: preoperative day; DO: postoperative day; D: postoperative day; H1-H20: the first 20 postoperative hours

The vital signs of the patient were stable before the operation (shown in Fig. 1). The intervention was accomplished under general anesthesia. During the operation, a catheter was inserted into the uterine cavity, and 20 mL of physiological saline was injected without obvious resistance, indicating that the fallopian tube was unobstructed. The cervix was dilated to 8.5 mm and a 8 mm-sheathed hysteroscopic morcellator was inserted into the uterus. Hysteroscopic inspection showed five endometrial polyps. The largest one was about 0.8*0.5 cm, while the smallest one was about 0.3*0.3 cm. All the polyps were cut to the base by the rotating movements of the inner blade, and the abraded fragments were aspirated and sent for pathological examination. The operation was successfully completed in about 8 min and the patient was sent to the ward for observation after surgery.

**Fig. 2:**
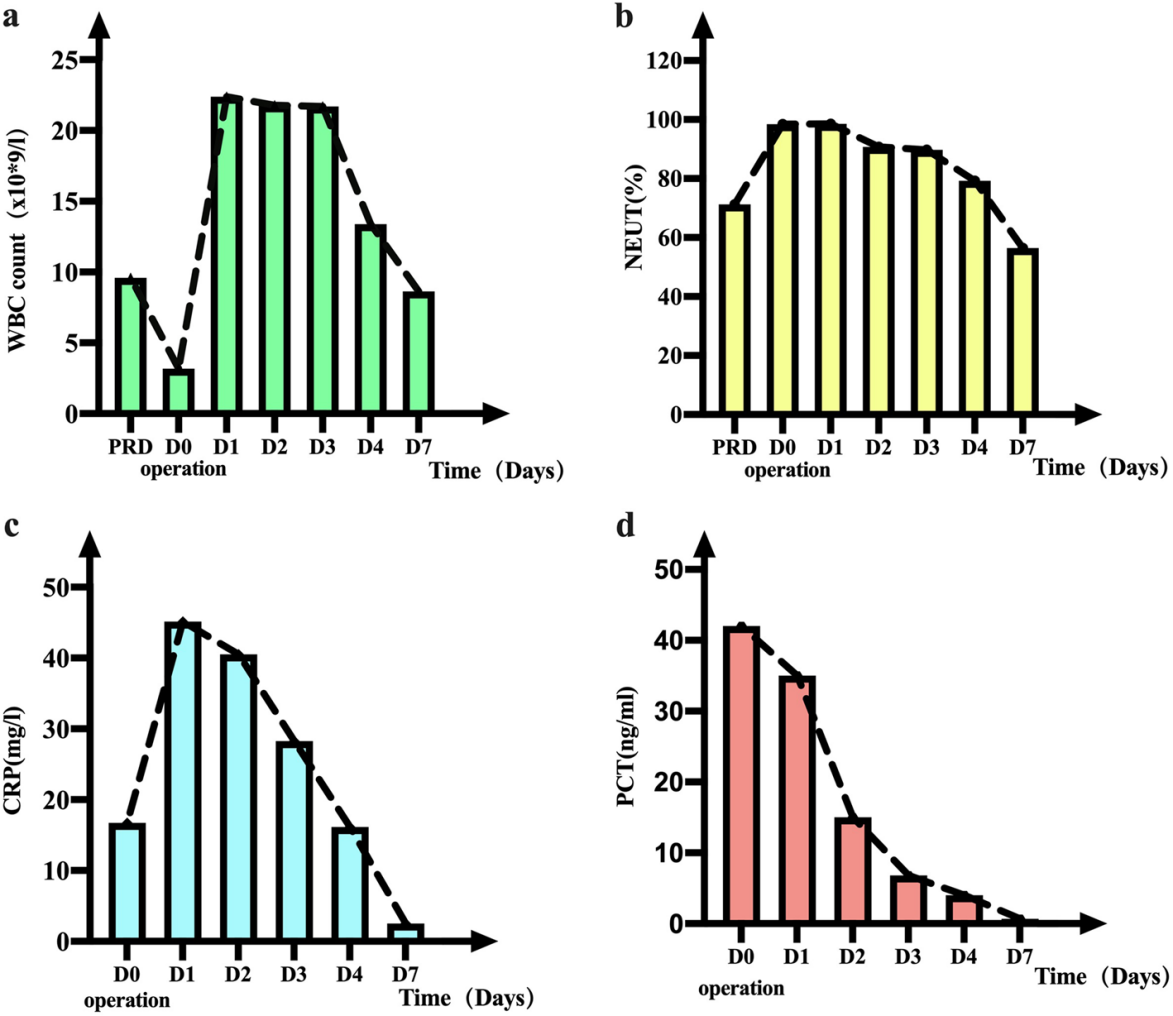
Changes of infection indicators during hospitalization. **a** Changes in the white blood cell count. **b** Changes in the proportion of neutrophils. **c** The development of hypersensitive C-reactive protein(CRP) values after Surgery. **d** The development of procalcitonin(PCT) values after Surgery. * PRD: preoperative day; DO: operation day; D: postoperative day

About 3 h after surgery, the patient developed mild lower abdominal pain with elevated body temperature (up to 38.7 °C)、heart rate (up to 92–120 bpm) and hypotension (about 72–83/35-54 mmHg) (shown in Fig. 1). Laboratory examination showed that white blood cells decreased to 3.18 × 10^9^/L (up to 22.39 × 10^9^/L later), the proportion of neutrophil increased (up to 98.5%), and the index of infection increased significantly, including hypersensitive C-reactive protein (up to 45.13 mg/l), procalcitonin (up to 42 ng/ml), interleukin (up to 3120 pg/ml) (shown in Fig. 2). Combined with the clinical manifestations and laboratory examination, the patient was considered to have postoperative septic shock. Then she was transferred to Intensive Care Unit (ICU) for further treatment.

**Fig. 3:**
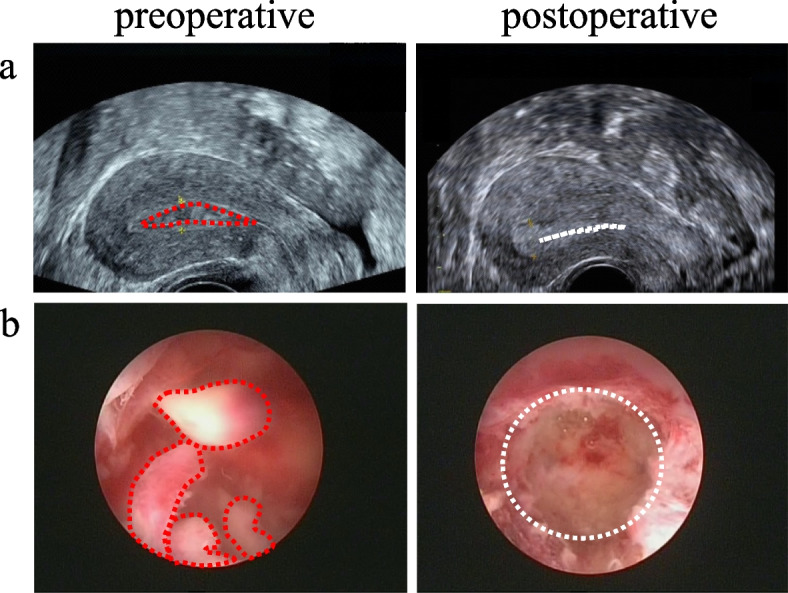
Ultrasound images of the uterus (**a**) and hysteroscopic view of the uterine cavity (**b**) before and after hysteroscopic polypectomy. * The red dotted circle represents the outline of the uterine cavity and endometrial polyps before polypectomy. The white dashed circle represents the outline of the uterine cavity after hysteroscopic polypectomy

**Fig. 4:**
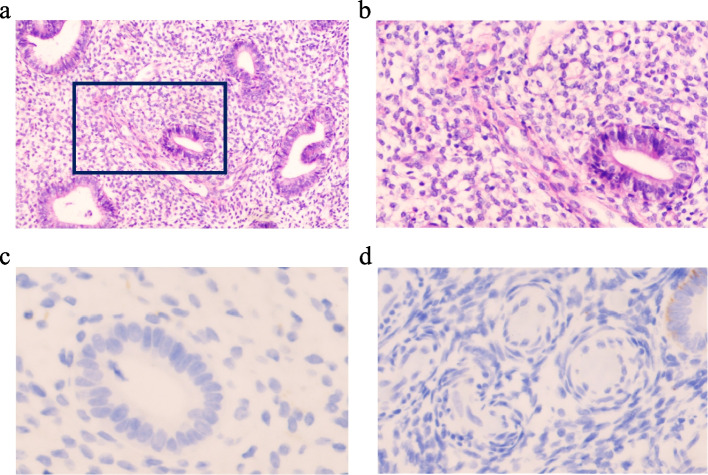
Pathological figures in the reported patient with endometrial polyps. **a**-**b** Endometrial polyps with hematoxylin and eosin(H&E), magnification(10 ×). The area indicated in a dark box is enlarged in the high power view on the right(40 ×). **c**-**d** Immunohistochemical staining for CD38(lower left) and CD138(lower right) reveales no plasma cell infiltration

The ICU physicians administered a combination of piperacillin tazobactam and tinidazole for antimicrobial treatment, while epinephrine was given for vasoconstrictive purposes. Additionally, fluid replacement and albumin supplementation were provided. After treatment, the patient's condition was gradually relieved, which was characterized by stable recovery of vital signs, gradual decline of white blood cells and infection indicators. At this moment, the blood culture result was negative. So, she was returned to Gynecology ward on the 3rd day after operation. After antibiotic therapy for 10 days, abdominal ultrasound was performed and showed a small amount of effusion. The results of white blood cells and various infection indicators were normal before discharged. Ultrasound imaging and hysteroscopic view were shown in Fig. 3. Histopathologic examination of Eps was shown in Fig. 4.

## Discussion

Our study was the first case to report a septic shock, after the hysteroscopic polypectomy with the tissue removal system. Different kinds of tissue removal systems share the same structural design and operating principle with others. All tissue removal devices use mechanical energy to simultaneously cut and aspirate tissue and the shaver blades consist of an outer sheath and an inner hollow tube with windows for simultaneous suction and cutting. Tissue removal system has advantages compared with conventional electroresection, being able to control the cutting depth by wrapping around the inner rotating blade with outer sheath, which plays an important role in the protection for endometrium. This technique has very promising features [8], making the operation faster, easier and with low potential complication rates [9, 10]. Therefore, it is meaningful to improve the clinicians' understanding of complications when using this system through this case.

Before the surgery, six courses of anti-inflammatory treatment were performed on the patient's vaginitis. After the routine vaginal examination indicated the absence of vaginitis, she was prepared for endometrial polypectomy. The reusable surgical instrument blade was sterilized. Aseptic procedures were also strictly followed during the whole operation. Therefore, postoperative complications caused by contamination of surgical instruments were not considered. Endometritis might not be excluded in this patient who failed in trying to conceive for more than half a year. However, immunohistochemical staining of the postoperative specimens revealed no positive staining for CD38 or CD138 (shown in Fig. 4), which might not be supposed chronic endometritis. Therefore, the available evidence was inadequate to propose that the patient was afflicted with chronic endometritis. Although no signs of vaginitis were identified in the preoperative leucorrhea routine detection, but this patient was prone to recurrent vaginitis probably due to the impaired immune response. So, the dysbiosis in vaginal microbiota or imbalance in vaginal microbiota microenvironment might not be excluded.

Using mechanical energy to cut tissue is the most significant feature of tissue removal system, indicating a potential disadvantage is the lack of high-frequency electrocoagulation possibilities. In other words, it is unable to cauterize blood vessels through thermal energy during the removal of lesions. It remains to be explored whether the slow closure of blood vessels provides an opportunity for pathogens to enter vascular system, thus leading to an increased risk of surgical infection. During the cutting process, the pathogens may spread through the unclosed blood vessels, thus having the possibility of inducing infectious shock. In this case report, the possibility of Gram-positive bacterial (in vivo) infection was considered according to the laboratory test indicators and successful empirical antibiotic treatment although the blood culture was negative. Therefore, for patients with a history of repeated vaginitis or poor body resistance, hysteroscope with tissue removal system should be carefully or not chosen for avoiding the risk of serious infection.

In conclusion, the purpose of this case report is to remind clinicians to pay attention to the selection and use of hysteroscope with tissue removal system, especially for patients with a history of repeated vaginitis. In addition, early identification of septic shock and timely application of antibiotic treatment are crucial, which can improve the prognosis of patients and avoid more serious outcomes.

## Data Availability

All data for the case reports are available in this manuscript.
